# Peripheral Dopamine Controlled by Gut Microbes Inhibits Invariant Natural Killer T Cell-Mediated Hepatitis

**DOI:** 10.3389/fimmu.2018.02398

**Published:** 2018-10-17

**Authors:** Rufeng Xue, Huimin Zhang, Jun Pan, Zhiwei Du, Wenjie Zhou, Zhi Zhang, Zhigang Tian, Rongbin Zhou, Li Bai

**Affiliations:** ^1^Division of Molecular Medicine, Hefei National Laboratory for Physical Sciences at Microscale, CAS Key Laboratory of Innate Immunity and Chronic Disease, School of Life Sciences, University of Science and Technology of China, Hefei, China; ^2^Key Laboratory of Brain Function and Disease of Chinese Academy of Science, Department of Biophysics and Neurobiology, University of Science and Technology of China, Hefei, China; ^3^Innovation Center for Cell Signaling Network, Hefei, China

**Keywords:** dopamine, D1-like receptors, hepatitis, iNKT, gut microbes

## Abstract

Neurotransmitters have been shown to regulate immune responses, and thereby are critically related to autoimmune diseases. Here we showed that depletion of dopaminergic neurons significantly promoted activation of hepatic iNKT cells and augmented concanavalin A (Con A)-induced liver injury. The suppressive effect of dopamine on iNKT cells was mediated by D1-like receptor-PKA pathway. Clearance of gut microbiota by antibiotic cocktail reduced synthesis of dopamine in intestines and exacerbated liver damage, and that could be restored by recovery of gut microbiota or replenishment of D1-like receptor agonist. Our results demonstrate that peripheral dopamine controlled by gut microbes inhibits IL4 and IFNγ production in iNKT cells and suppresses iNKT cell-mediated hepatitis. Together, we propose a gut microbe-nervous system-immune system regulatory axis in modulating autoimmune hepatitis.

## Introduction

Recent studies indicate a crosstalk between nervous system and immune system. Neurotransmitters, neuropeptides, cytokines and their receptors are main mediators in the neuro-immune network. Dopamine, a critical transmitter, has been shown to regulate immune responses in periphery, and has been related to tumor immunity and several autoimmune diseases, including inflammatory bowel diseases, multiple sclerosis and rheumatoid arthritis (1–3). Dopamine receptors have been detected in both innate immune cells and adaptive immune cells. According to the downstream signaling, these receptors can be classified into two subclasses (4). D1-like dopamine receptors (DRD) including DRD1 and DRD5 activate adenylyl cyclase, whereas D2-like receptors including DRD2, DRD3, and DRD4 inhibit adenylyl cyclase. These receptors show distinct affinity for dopamine: DRD3 > DRD5 > DRD4 > DRD2 > DRD1 (5). Thus, dopamine displays complex regulatory effect on immune responses, depending on dopamine concentration, subtype of receptors and type of immune cells. As an important immune regulator, peripheral dopamine is mainly produced by autonomic nervous system, gut epithelial cells, and immune cells including dendritic cells, regulatory T cells, B cells, and macrophages (5). About 50% dopamine is produced in gastrointestinal tract by enteric neurons and intestinal epithelial cells, and thus results in higher level of dopamine in hepatic portal vein (6). Crosstalk between gut and liver has been demonstrated by plenty of studies. Progression of liver diseases is associated with molecules and cells derived from gut (7). The contributions of gut derived dopamine to liver immunity and autoimmune liver diseases remain to be elucidated.

As the most abundant innate lymphocytes in liver, invariant NKT (iNKT) cells play crucial roles in liver immunity and are critically linked to liver diseases (8). Different from conventional T cells, iNKT cells are tissue-resident cells and contribute to the first line of body defense. Upon stimulation, they release large amount of Th1 and Th2 cytokines, and bridge innate and adaptive immunity. Several factors modulating iNKT cell functions have been characterized, including antigens, cytokines, antigen presenting cells (APCs), and metabolites (9–12). Interestingly, nervous system has also been suggested to regulate the behavior of iNKT cells *in vivo* (13). During hepatocyte regeneration, sympathetic nervous system induces expansion of NKT cells (14). Additionally, norepinephrine inhibits apoptosis of NKT cells and restores hepatic NKT cell numbers in ob/ob mice (15). These findings demonstrate a link between nervous system and iNKT cells. However, the influences of dopamine on hepatic iNKT cell functions and iNKT cell related liver diseases are still unclear.

Here, we demonstrate that dopamine plays an important role in suppressing autoimmune hepatitis. Depletion of dopaminergic neurons using 1-methyl-4-phenyl-1,2,3,6-tetrahydropyridine (MPTP) significantly augmented the concanavalin A (Con A)-induced hepatitis. Dopamine inhibited IL4 and IFNγ production in iNKT cells through D1-like receptor-PKA pathway, and thus suppressed the iNKT cell-mediated liver damage. Moreover, synthesis of peripheral dopamine was controlled by gut microbes. Clearance of gut microbes using antibiotics reduced dopamine synthesis in guts, and consequently promoted Con A-induced liver injury. Restoring dopamine synthesis via transferring gut microbes or replenishing D1-like receptor agonist ameliorated the liver damage in antibiotics-treated mice. Our study proposes a regulatory axis from gut microbes to neurotransmitter and then to autoimmune hepatitis.

## Materials and methods

### Mice and treatment

WT mice were purchased from the Beijing Vital River Laboratory Animal Technology. *J*α*18*^−/−^ mice and *V*α*14 Tg.cxcr6*^*gfp*/+^ mice have previously been described and were provided by Dr. Bendelac. All male mice used were in C57BL/6 background and between 6 and 10 weeks of age, and were maintained under pathogen-free conditions. All animal procedures were approved by the USTC Institutional Animal Care and use Committee.

To investigate the influence of A68930 on iNKT cell functions *in vivo*, mice were injected intraperitoneally with α-GalCer (2 μg/mouse, Avanti Polar Lipids, Alabama) and A68930 (8 mg/kg, Sigma-Aldrich, Munich, Germany), 4 h before tissue collection. For the control group, mice were injected with α-GalCer only. To induce hepatitis, Con A (Sigma-Aldrich, Munich, Germany) was injected into the mice at a concentration of 15 mg/kg with or without A68930 or A77636 (8 mg/kg) (Sigma-Aldrich, Munich, Germany). Tissue samples and serum were collected after injecting Con-A for 12 h. To delete dopaminergic neurons *in vivo*, MPTP (20 mg/kg, Sigma-Aldrich, Munich, Germany) or PBS buffer as control was injected intraperitoneally for 4 times with 2 h intervals. Twenty-four hours after the last injection, these mice were injected with Con A with or without A77636 to induce liver damage. Livers were fixed in 4% paraformaldehyde, embedded in paraffin, sectioned, and stained with hematoxylin and eosin for histological examination. Alanine aminotransferase and aspartate aminotransferase in serum were measured by a Chemray-240 Automated Chemistry Analyzer (Rayto, Shenzhen, China).

### Clearance and restoration of gut microbiota

Four antibiotics in drinking water were used to treat mice for 2 weeks: vancomycin (1 g/L), streptomycin (2 g/L), metronidazole (2 g/L), and ampicillin (2 g/L). To restore the gut microbiota, antibiotics-treated mice were co-housed with age-matched normal mice for 4 weeks without antibiotics treatment.

### Stimulation of iNKT cells

iNKT cells, gating as GFP^hi^ cells, were sorted from livers of *V*α*14 Tg.cxcr6*^*gfp*/+^ mice (Figure S1), and were stimulated with α-GalCer-pulsed (1 μg/ml) RBL.CD1d cells or plate-coated anti-CD3 (10 μg/ml, Biolegend, San Diego, California) plus anti-CD28 (1 μg/ml, Biolegend, San Diego, California) antibodies in the presence of indicated reagents for 16 h. Cytokines in supernatants were measured by cytometric bead array. To measure intracellular cyclic adenosine monophosphate (cAMP), cells were stimulated with phorbol myristate acetate (PMA, 50 ng/ml) plus ionomycin (1 μM) for 4–6 h.

### Intracellular cytokine and camp staining

After surface staining, cells were fixed with 4% paraformaldehyde (Sigma-Aldrich, Munich, Germany) and permeabilized with PBS buffer containing 0.1% saponin (Sigma-Aldrich, Munich, Germany) and 0.5% bovine serum albumin (BSA, Sigma-Aldrich, Munich, Germany). Then, cells were stained with antibodies against intracellular cytokines or cAMP. Anti-TCRβ (H57-597), anti-IFNγ (XMG1.2), and anti-IL4 (11B11) were purchased from Biolegend (San Diego, California). Anti-cAMP was purchased from Abcam (Cambridge, England). PBS57-CD1d tetramer was provided by the NIH Tetramer Core Facility. Cells were analyzed with a FACSVerse flow cytometer (BD Biosciences, Franklin Lakes, NJ) and data was analyzed with FlowJo 7.6 software (Tree star, Ashland, Oregon).

### High-performance liquid chromatography (HPLC)

To measure the dopamine in portal vein, portal venous blood was collected from control or MPTP treated mice. Two hundred fifty microliter serum was mixed with 125 μl 2 M perchloric acid (HCLO_4_), and then was centrifuged at 5,000 g for 10 min. The supernatant was collected and injected into the HPLC system (Antec Scientific, Zoeterwoude, Netherlands) for dopamine analysis. Dopamine standard was used to determine the concentration.

### Real-time PCR

Total RNA was extracted from stimulated cells with ReliaPrep™ RNA Cell Miniprep System (Promega, Fitchburg, Wisconsin). cDNA was synthesized from total RNA using Reverse Transcription System (Promega, Fitchburg, Wisconsin). Quantitative PCR was performed using GoTaq qPCR Master Mix (Promega, Fitchburg, Wisconsin). *Actin* was used as an internal control gene. The primer sequences used were as follows:

*drd1* F 5′ GGATGTGCATCGAGGTGAATG; *drd1* R 5′CGATGAGGCACAGCTCATT 3′; *drd2* F 5′ CAGATGCTTGCCATTGTTCT 3′; *drd2* R 5′ CAGCAGTGCAGGATCTTCAT 3′; *drd3* F 5′ GTGGCTCGGGGCCTTCATTG 3′; *drd3* R 5′ GGGCACTGTTCACGTAGCCA 3′; *drd4* F 5′ GTGTTGGACGCCTTTCTTCG 3′; *drd4* R 5′ GGGTTGAGGGCACTGTTGA 3′; *drd5* F 5′ CTGCGAGCATCCATCAAG 3′; *drd5* R 5′ CACAAGGGAAGCCAGTCC 3′; *IL4* F 5′ATGGAGCTGCAGAGACTCTT 3′; *IL4* R 5′ AAAGCATGGTGGCTCAGTAC 3′; *Ifng* F 5′ ATGAACGCTACACACTGCATC 3′; *Ifng* R 5′ CCATCCTTTTGCCAGTTCCTC 3′; *Th* F 5′ GACAGTCCTCACACCATCCG 3′; *Th* R 5′ GACAGTCCTCACACCATCCG 3′.

### Western blot

Cells or tissues were harvested and lysed with sample buffer and boiled for 10 min. Proteins were separated by electrophoresis and detected by western blot. Antibodies against CREB, pSer133-CREB, IκBα, pSer32-IκBα, TH, and Actin were purchased from Cell Signaling Technology (Danvers, Massachusetts), Sigma-Aldrich (Munich, Germany), Abcam (Cambridge, England), or Proteintech (Chicago, Illinois).

### Bacterial genomic DNA extraction and amplification of 16S rRNA

Fresh feces were collected from the experimental mice, bacterial genomic DNA was extracted using the YuanPingHao Bio stool kit (Beijing, China). The amounts of different gut bacteria were measured by qPCR using primers specific for their 16S rRNA as previously described (16). Group-specific primers were used as follows: *Eubacterium rectale-Clostridium coccoides* (Erec), UniF338, 5′ACTCCTACGGGAGGCAGC 3′, C.cocR491, 5′GCTTCTTTAGTCAGGTACCGTCAT 3′; *Bacteroides* (Bact), BactF285, 5′GGTTCTGAGAGGAGGTCCC 3′, UniR338, 5′ GCTGCCTCCCGTAGGAGT 3′; *mouse intestinal Bacteroides* (MIB), Uni516F, 5′ CCAGCAGCCGCGGTAATA 3′, MIBR677, 5′ CGCATTCCGCATACTTCTC 3′; *Enterobacteriaceae* (Ent), 515F, 5′GTGCCAGCMGCCGCGGTAA 3′, 826R, 5′GCCTCAAGGGCACAACCTCCAAG 3′; Eubacteria (All bacteria), UniF340, 5′ACTCCTACGGGAGGCAGCAGT 3′, UniR514, 5′ATTACCGCGGCTGCTGGC3′.

### Statistical analyses

Error bars represent SEM. Statistical analyses were performed using student's *t*-test (GraphPad Software). ^*^p < 0.05, ^**^p < 0.01, and ^***^p < 0.001 were considered statistically significant.

## Results

### Depletion of dopaminergic neurons augments con a-induced liver injury

**Figure 1:**
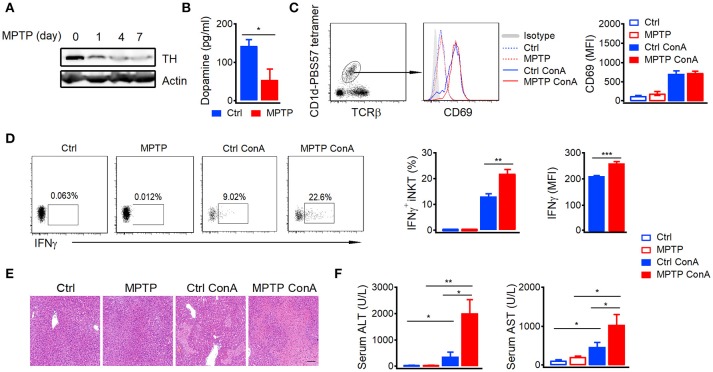
Depletion of dopaminergic neurons promotes Con A-induced liver injury.**(A)** Expression of TH in brains of mice injected with MPTP (20 mg/kg) for indicated times. **(B)** Dopamine in portal vein of control mice and MPTP treated mice (24 h later). **(C,D)** CD69 expression **(C)**, percentages of IFNγ^+^ hepatic iNKT cells and mean fluorescence intensity of IFNγ **(D)** in mice injected with Con A (15 mg/kg) with or without MPTP (20 mg/kg) pretreatment. **(E)** Hematoxylin and eosin staining of liver tissues from mice described in **(C,D)**. Bar, 100 μm. **(F)** ALT and AST in serum of mice described in **(C,D)**. *n* = 5 mice per group. Error bars represent SEM. ^*^P < 0.05, ^**^P < 0.01, ^***^P < 0.001. MPTP, 1-methyl-4-phenyl-1,2,3,6-tetrahydropyridine; MFI, mean fluorescent intensity; ConA, Concanavalin A.

Previous studies indicate that large amount of peripheral dopamine is detected in hepatic portal vein (6). To demonstrate the role of dopamine in autoimmune hepatitis, we depleted peripheral dopamine by injecting mice with dopaminergic neuron-specific neurotoxin MPTP (17). MPTP efficiently depleted dopaminergic neurons as indicated by reduced expression of tyrosine hydroxylase, a key enzyme for dopamine biosynthesis, in brains (Figure 1A). Moreover, the concentration of dopamine in portal vein (Figure 1B) and mRNA of tyrosine hydroxylase in gut (Figure S2) were also significantly reduced by MPTP. It is well-known that iNKT cells are the main mediators in Con A-induced acute autoimmune hepatitis (18). Although depletion of dopaminergic neurons by MPTP did not influence the Con A-induced expression of CD69 in hepatic iNKT cells (Figure 1C), it significantly elevated their IFNγ production (Figure 1D). In agreement with previous findings that iNKT cells and IFNγ play important roles in the development of Con-A induced hepatitis (19, 20), exacerbated hepatocyte necrosis (Figure 1E) and increased alanine aminotransferase (ALT) as well as aspartate aminotransferase (AST; Figure 1F) were detected in MPTP treated mice after Con-A injection. These results demonstrated severer Con A-induced liver injury in MPTP treated mice than in control mice, suggesting a role of dopamine in suppressing autoimmune hepatitis.

### Dopamine inhibits IL4 and IFNγ production in iNKT cells

**Figure 2:**
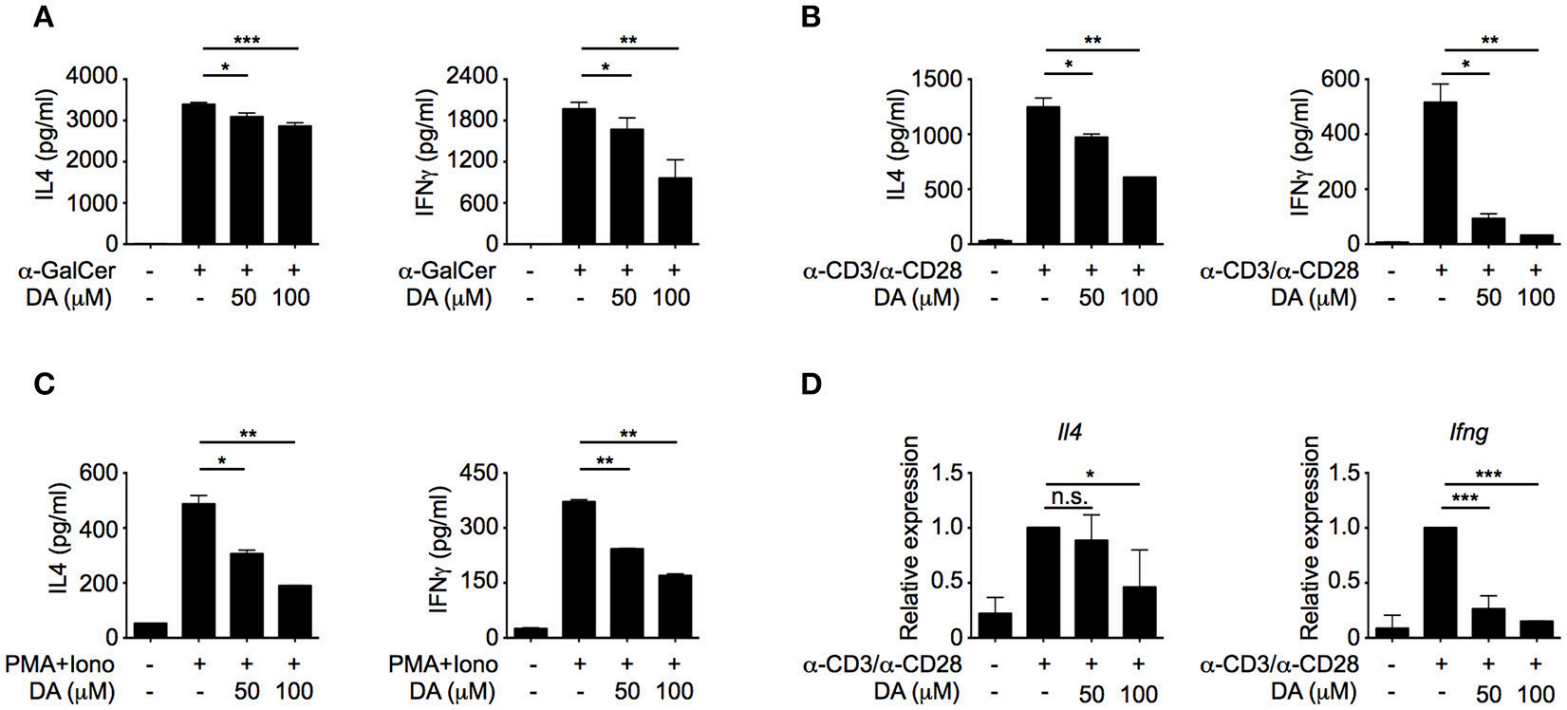
Dopamine inhibits production of IL4 and IFNγ in iNKT cells. **(A–C)** IL4 and IFNγ production in iNKT cells activated by α-GalCer-pulsed RBL.CD1d cells **(A)**, anti-CD3 plus anti-CD28 **(B)**, and PMA plus ionomycin **(C)** in the absence or presence of different doses of dopamine. Data are representative of three independent experiments. **(D)** mRNA levels of *Il4* and *Ifng* in iNKT cells activated by anti-CD3 plus anti-CD28, in the absence or presence of different doses of dopamine. Data are representative of three independent experiments. Error bars represent SEM. ^*^P < 0.05, ^**^P < 0.01, ^***^P < 0.001. DA, dopamine; PMA, phorbol myristate acetate; Iono, ionomycin.

To investigate whether dopamine could directly regulate iNKT cell functions, different doses of dopamine were added to iNKT cells that were activated by α-GalCer-pulsed RBL.CD1d cells. Dopamine inhibited both IL4 and IFNγ production from iNKT cells in a dose-dependent manner (Figure 2A). Similar results were obtained when NKT cells were activated by plate-coated anti-CD3 plus anti-CD28 antibodies (Figure 2B) or by PMA plus ionomycin bypassing T cell receptor (TCR) signaling (Figure 2C). These results indicate that dopamine could inhibit production of cytokines in iNKT cells in a TCR-independent manner. Next, we investigated whether dopamine inhibited IL4 and IFNγ production at transcriptional level. Reduced *Il4* and *Ifng* mRNA were detected in dopamine treated cells, suggesting a transcriptional inhibition by dopamine (Figure 2D). To exclude the possibility that diminished cytokine production was caused by cell death, we measured lactate dehydrogenase release in culture medium. Lactate dehydrogenase is a cytosolic enzyme and is released into culture medium when the plasma membrane is damaged. In our studies, dopamine did not increase the lactate dehydrogenase in medium, indicating normal cell viability after dopamine treatment (Figure S3A).

### Dopamine inhibits IL4 and IFNγ production in iNKT cells through D1-like receptors

**Figure 3:**
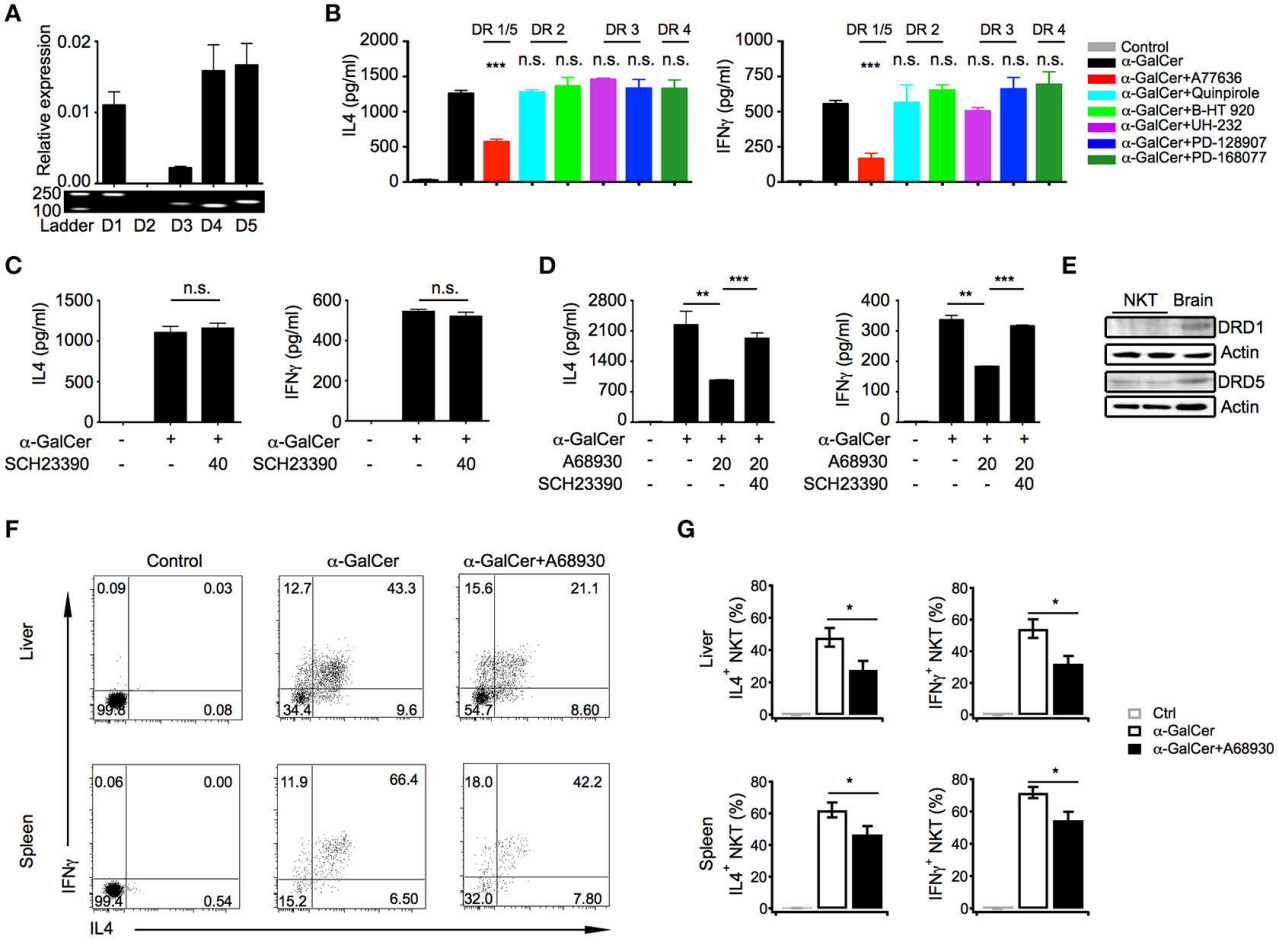
Dopamine inhibits cytokine production in iNKT cells through D1-like receptors. **(A)** mRNA level and fragments of various dopamine receptors in iNKT cells. **(B)** Influences of indicated selective receptor agonists (20 μM) on production of IL4 and IFNγ in iNKT cells. iNKT cells were activated by α-GalCer-pulsed RBL.CD1d cells. **(C)** Influences of SCH23390, antagonist of D1-like receptors, on IL4 and IFNγ production in iNKT cells. **(D)** Influences of A68930, agonist of D1-like receptors, on IL4 and IFNγ production in iNKT cells pretreated with or without SCH23390 for 3 h. **(E)** Protein levels of DRD1, DRD5, and β-actin in iNKT cells. Brain was used as positive control. Data are representative of more than three independent experiments. **(F)** Intracellular staining of IL4 and IFNγ in hepatic and splenic iNKT cells after injecting α-GalCer (2 μg/mouse) with or without A68930 (8 mg/kg) for 4 h. In negative controls, mice were injected with PBS buffer (*n* = 8 mice per group). **(G)** Percentages of IL4^+^ and IFNγ^+^ iNKT cells as in **(F)**. Error bars represent SEM. ^*^P < 0.05, ^**^P < 0.01, ^***^P < 0.001. DR, dopamine receptor.

Dopamine exerts its effects through five distinct receptors (DRD1 to DRD5). Except for DRD2, mRNA of other four receptors were detected in iNKT cells, including DRD1, DRD5, DRD3, and DRD4, although DRD3 level was relatively low (Figure 3A). To determine which receptor was involved in the suppressive effects of dopamine, selective receptor agonists had been used to activate distinct receptor, respectively. Interestingly, dopamine D1-like receptor agonist A77636, activating DRD1, and DRD5, significantly inhibited IL4 and IFNγ production, whereas agonists of DRD2, DRD3, and DRD4 showed no effect on cytokine production (Figure 3B). To further prove that dopamine inhibited cytokine production through D1-like receptors, we blocked D1-like receptors with antagonist SCH23390. Although SCH23390 did not influence IL4 and IFNγ production in iNKT cells (Figure 3C), it abrogated the inhibitory effect of D1-like receptor agonist A68930 (Figure 3D). These results confirmed the specificity of agonist for D1-like receptors. All D1-like receptor agonists showed no influence on iNKT cell viability (Figure S3B). However, there is no selective agonist that could distinguish DRD1 from DRD5. Although mRNA of DRD1 and DRD5 were detected at similar level in iNKT cells, DRD5 rather than DRD1 was clearly detected at protein level (Figure 3E). Taken together, dopamine might inhibit cytokine responses in iNKT cells through D1-like receptors.

In order to investigate the role of dopamine in regulating iNKT cell functions *in vivo*, α-GalCer was injected into mice with or without D1-like receptor agonist A68930. A68930 significantly reduced percentages of IL4^+^ and IFNγ^+^ iNKT cells in livers and in spleens (Figures 3F,G). These results confirmed suppressive effect of dopamine on iNKT cell functions *in vivo*.

### Dopamine inhibits iNKT cell functions via cAMP-PKA pathway

**Figure 4:**
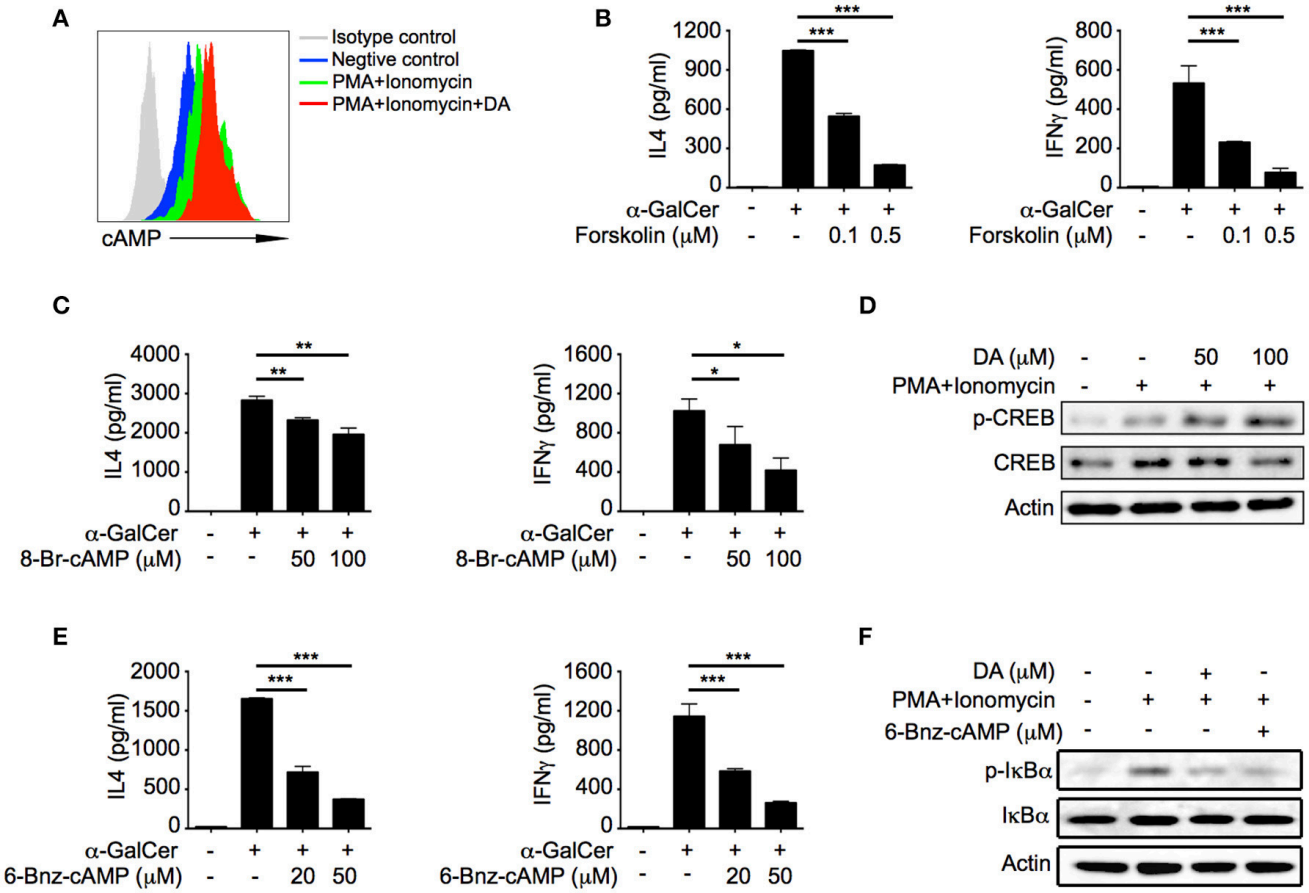
Dopamine inhibits iNKT cell functions via cAMP-PKA pathway. **(A)** cAMP level in iNKT cells in the presence of indicated reagents. Gray line, isotype control; blue line, medium; green line, PMA plus Ionomycin; red line, PMA plus ionomycin plus dopamine (50 μM). **(B,C)** IL4 and IFNγ production in α-GalCer activated iNKT cells in the presence of different doses of forskolin **(B)** and 8-Br-cAMP **(C)**. **(D)** Immunoblot analysis of p-CREB, CREB, and β-actin in iNKT cells activated by PMA plus ionomycin in the absence or presence of dopamine. **(E)** IL4 and IFNγ production in α-GalCer activated iNKT cells in the presence of different doses of 6-Bnz-cAMP. **(F)** Immunoblot analysis of p-IκBα, IκBα, and β-actin in iNKT cells activated by PMA plus ionomycin in the absence or presence of dopamine (50 μM) or 6-Bnz-cAMP (50 μM). Data are representative of three independent experiments. Error bars represent SEM. ^*^P < 0.05, ^**^P < 0.01, ^***^P < 0.001. PMA, phorbol myristate acetate; DA, dopamine.

D1-like receptors are coupled to G_s_, which activates adenylyl cyclase and promotes production of cAMP (21). Consistent with previous studies, dopamine significantly increased cAMP level in activated iNKT cells (Figure 4A). cAMP has been shown to negatively regulate cytokine production in a variety of cells (22). It is possible that suppressive effect of dopamine was due to elevation of cAMP in iNKT cells. We increased intracellular cAMP concentration in iNKT cells with forskolin, a specific activator of adenylyl cyclase, and observed significant reduction of IL4 and IFNγ in a dose dependent manner (Figure 4B). Furthermore, cAMP analog 8-Br-cAMP inhibited IL4 and IFNγ production as well (Figure 4C). These results demonstrated that increased intracellular cAMP was responsible for the inhibitory effect of dopamine on iNKT cell functions. Protein kinase A (PKA) is a cAMP-dependent protein kinase, which has been previously shown to inhibit T cell functions (23). In our studies, we detected phosphorylation of CREB-S133 (Figure 4D), a substrate of PKA, in dopamine treated cells, which indicated activation of PKA. To investigate whether dopamine inhibited production of cytokines in iNKT cells via activating PKA, we measured cytokine responses in the presence of 6-Bnz-cAMP, a specific activator of PKA. 6-Bnz-cAMP significantly inhibited IL4 and IFNγ production (Figure 4E). These results confirm that dopamine inhibits iNKT cell functions by activating cAMP-PKA pathway. Again, forskolin, 8-Br-cAMP, and 6-Bnz-cAMP did not increase the lactate dehydrogenase in medium (Figure S3C). It has been reported that PKA could inhibit TCR signaling via activating CSK (24, 25). However, dopamine suppressed cytokines in iNKT cells when TCR proximal signaling was bypassed by PMA plus ionomycin (Figure 2C). Next, we investigated the influences of dopamine and PKA activator on signal pathways downstream TCR. Activation of NFκB was significantly inhibited by dopamine and 6-Bnz-cAMP, as indicated by reduced phosphorylation of IκBα (Figure 4F). It has been shown previously that activation of NFκB is important for IL4 and IFNγ production in iNKT cells (26). Together, our results demonstrate an inhibitory effect of dopamine on iNKT cell functions via repressing NFκB signal pathway.

### D1-like receptor agonists inhibit iNKT cell-mediated liver injury

**Figure 5:**
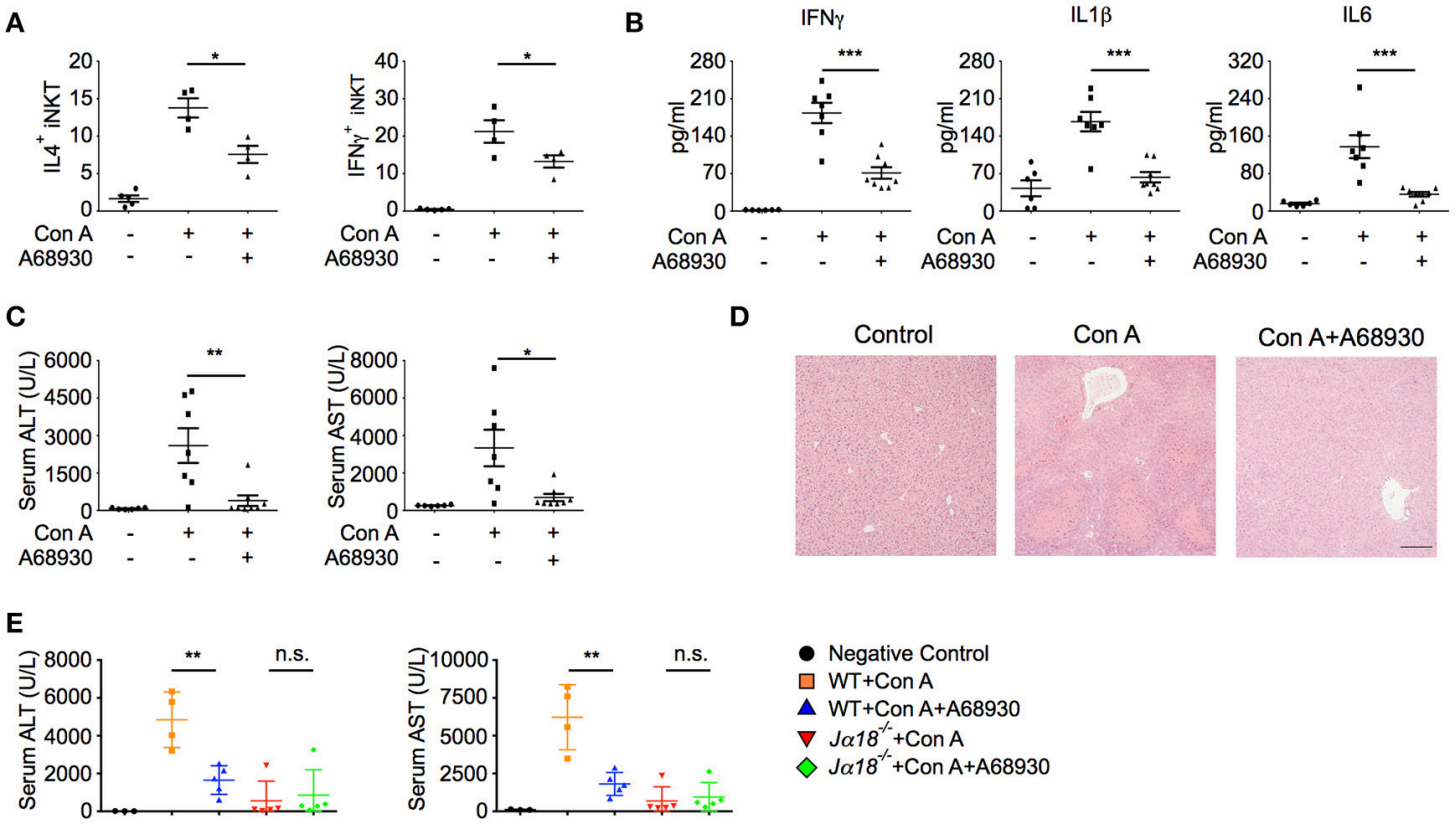
A68930 inhibits Con A-induced liver injury. **(A–C)** Percentages of IL4^+^ and IFNγ^+^ iNKT cells in livers (*n* = 4–5 mice per group), TNFα, IL1β, and IL6 in liver homogenates **(B)**, ALT and AST in serum **(C)** of mice injected with Con A (15 mg/kg) alone or with both A68930 (8 mg/kg) and Con A (*n* = 6–8 mice per group). **(D)** Hematoxylin and eosin staining of liver tissues from mice described in **(B)**. Bar, 100 μm. **(E)** ALT and AST in serum of WT mice and *J*α*18*^−/−^ mice treated with indicated reagents (*n* = 3–5 mice per group). Error bars represent SEM. ^*^P < 0.05, ^**^P < 0.01, ^***^P < 0.001. ConA, Concanavalin A; ALT, alanine transaminase; AST, aspartate aminotransferase.

In agreement with the suppressive effects of A68930 on cytokine production in iNKT cells (Figure 3D), it dramatically inhibited Con A-induced IL4 and IFNγ production in hepatic iNKT cells (Figure 5A). Consistently, Con A-induced liver injury was significantly prevented by A68930, as indicated by reduced pro-inflammatory cytokine production in liver (Figure 5B), diminished ALT and AST (Figures 5C), and ameliorated histological damage (Figure 5D). In iNKT cell deficient *J*α*18*^−/−^ mice, Con A caused much less liver damage than in WT mice, as indicated by the lower levels of ALT and AST (Figure 5E). These results are consistent with previous findings that iNKT cells are main mediators of Con A-induced liver injury. Importantly, A68930 only reduced ALT and AST in WT mice but not in *J*α*18*^−/−^ mice (Figure 5E). Therefore, the suppressive effect of A68930 on Con A-induced liver injury is attributed to its inhibitory effect on iNKT cell functions.

**Figure 6:**
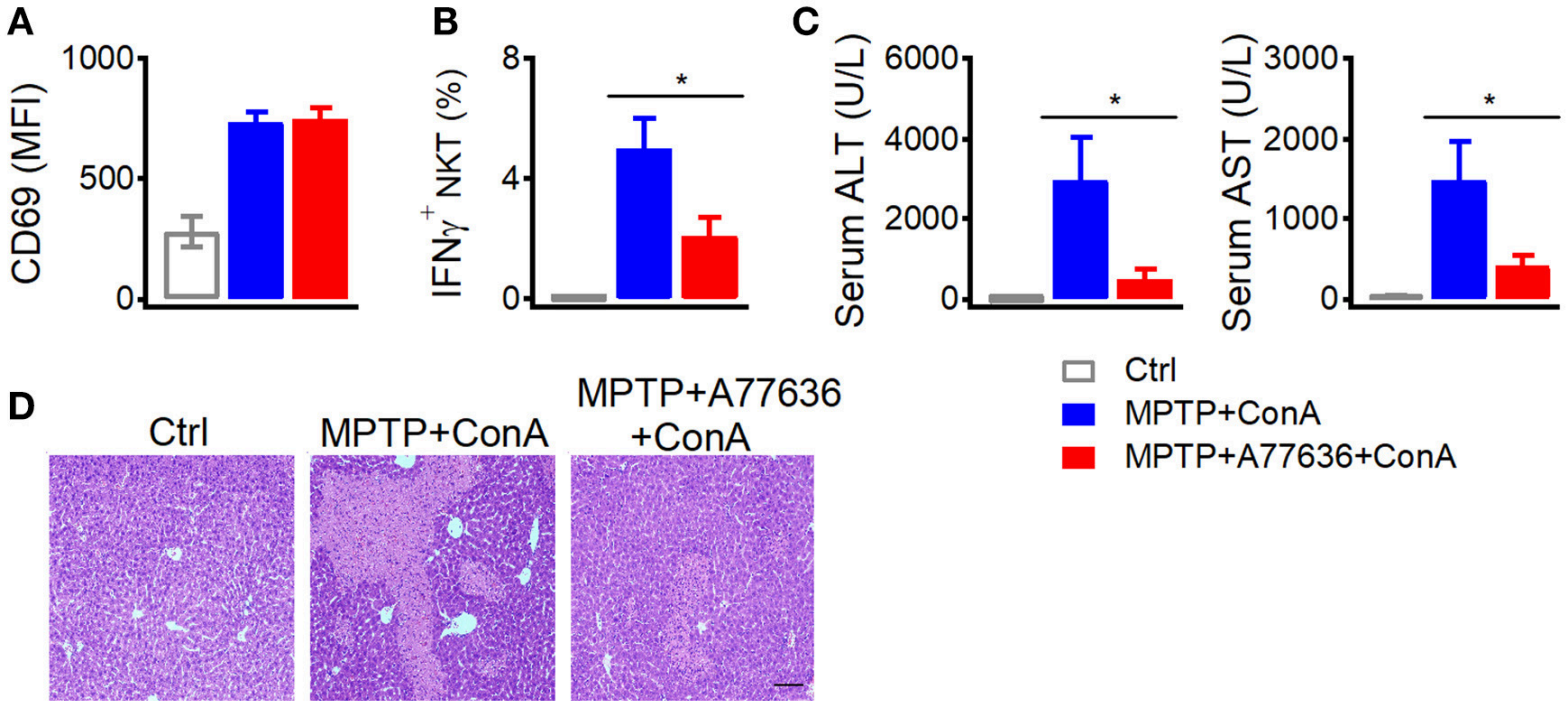
A77636 inhibits Con A-induced liver injury in MPTP treated mice. **(A–D)** Mean fluorescence intensity of CD69 in hepatic iNKT cells **(A)**, percentages of IFNγ^+^ hepatic iNKT cells **(B)**, ALT and AST in serum **(C)**, hematoxylin and eosin staining of liver tissues **(D)** from MPTP treated mice receiving Con A (15 mg/kg) or A77636 (8 mg/kg) plus Con A (*n* = 4–5 mice per group). Bar, 100 μm. Error bars represent SEM. ^*^P < 0.05. MPTP, 1-methyl-4-phenyl-1,2,3,6-tetrahydropyridine; MFI, mean fluorescent intensity; ConA, Concanavalin A; ALT, alanine transaminase; AST, aspartate aminotransferase.

Additionally, deficiency of dopamine caused severer liver damage after Con-A injection (Figure 1), which was abrogated by another D1-like receptor agonist A77636. In MPTP treated mice, A77636 showed no influence on Con-A induced CD69 expression (Figure 6A) but reduced Con-A induced IFNγ production in hepatic iNKT cells (Figure 6B), diminished serum ALT and AST (Figure 6C), and ameliorated histological damage (Figure 6D). These results further confirmed that the exacerbated liver injury in MPTP mice was due to the insufficient dopamine.

### Gut microbes promote dopamine synthesis and suppress con a-induced liver injury

**Figure 7:**
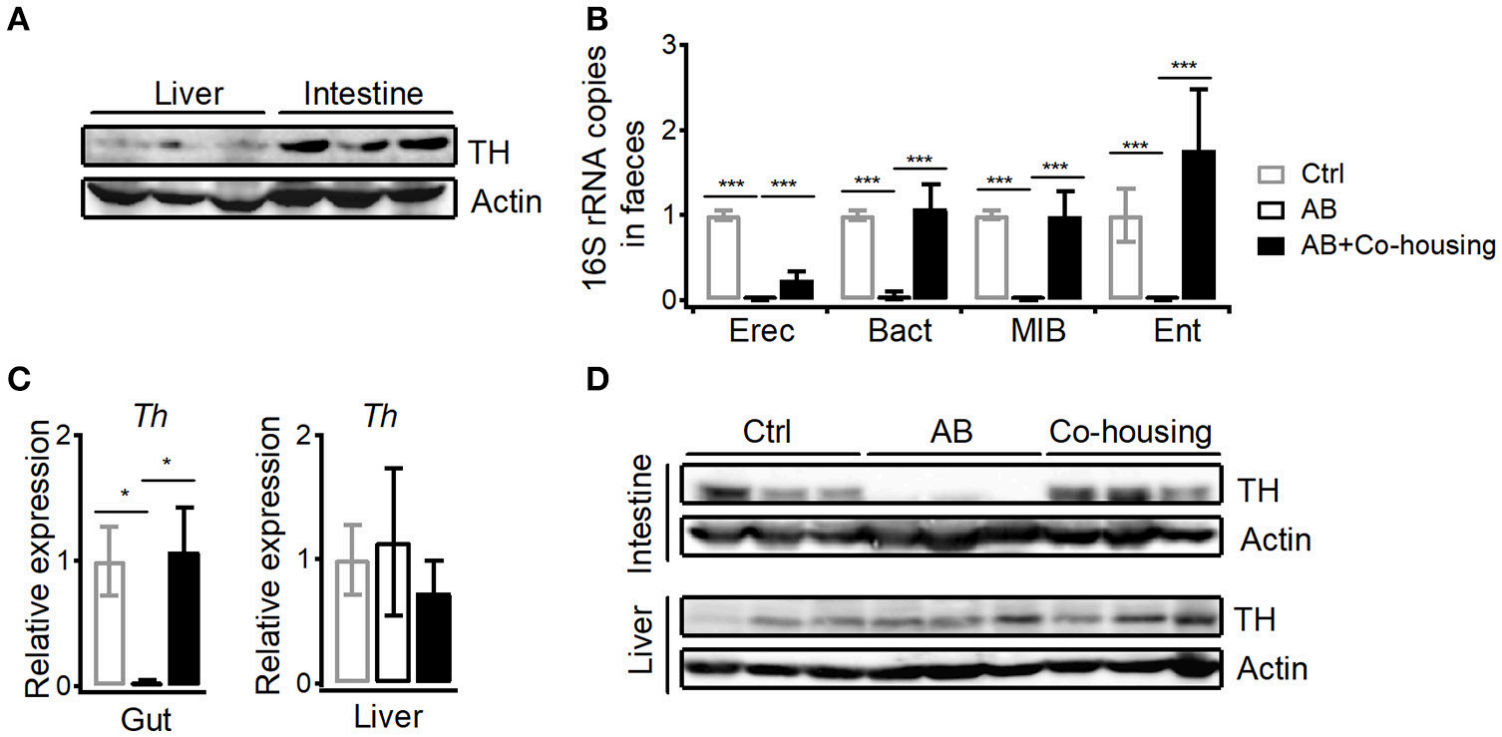
Gut microbes promote dopamine synthesis in small intestines. **(A)** Expression of TH in livers and in small intestines. Data are representative of three independent experiments. **(B)** 16S rRNA of four bacterial groups in feces of control mice, antibiotics-treated mice, and antibiotics-treated mice co-housed with normal mice (*Eubacterium rectale-Clostridium coccoides*, Erec; *Bacteroides*, Bact; *mouse intestinal Bacteroides*, MIB; *Enterobacteriaceae*, Ent). **(C,D)** mRNA **(C)** and protein **(D)** of TH in small intestines and in livers from mice described in **(B)**. *n* = 10–15 mice per group. Error bars represent SEM. ^*^P < 0.05, ^***^P < 0.001. TH, tyrosine hydroxylase; AB, antibiotic; MFI, mean fluorescent intensity.

**Figure 8:**
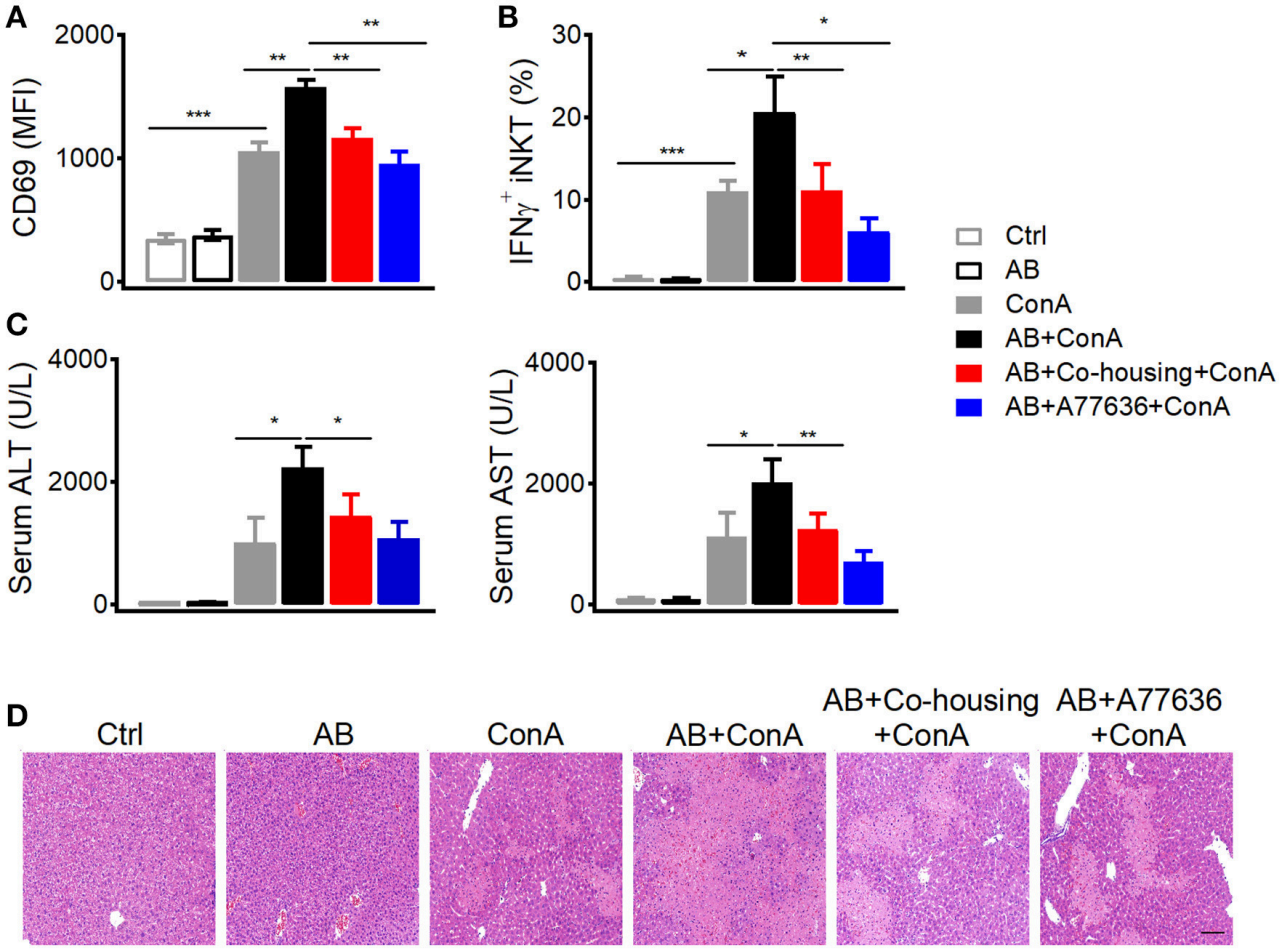
Gut microbes inhibit Con A-induced liver injury. **(A–D)** Mean fluorescence intensity of CD69 in hepatic iNKT cells **(A)**, percentages of IFNγ^+^ hepatic iNKT cells **(B)**, ALT and AST in serum **(C)**, hematoxylin and eosin staining of liver tissues **(D)** from indicated mice after Con A (15 mg/kg) or A77636 (8 mg/kg) plus Con A injection (*n* = 10–15 mice per group). Bar, 100 μm. Error bars represent SEM. ^*^P < 0.05, ^**^P < 0.01, ^***^P < 0.001. AB, antibiotic; MFI, mean fluorescent intensity; ConA, Concanavalin A; ALT, alanine transaminase; AST, aspartate aminotransferase.

In agreement with previous findings that peripheral dopamine mainly derives from gut (5, 6, 27), much higher level of tyrosine hydroxylase protein was detected in small intestines than in livers (Figure 7A). It has been shown that bacteria are able to either influence the production of neurotransmitters or generate many neurotransmitters directly including gamma-aminobutyric acid (GABA), norepinephrine (NE), and 5-hydroxytryptamine (5HT) (28). To investigate the influence of gut microbes on peripheral dopamine synthesis and hepatic iNKT cell functions, we cleared gut microbiota with antibiotic cocktail containing four antibiotics. Antibiotic cocktail successfully cleared major bacterial groups in feces, and co-housing antibiotics-treated mice with normal mice recovered gut microbiota (Figure 7B). Importantly, antibiotics significantly reduced tyrosine hydroxylase at mRNA level and protein level in small intestines, but showed no effect in livers (Figures 7C,D). Recovery of gut microbiota in antibiotics-treated mice restored expression of intestinal tyrosine hydroxylase (Figures 7C,D). These results indicate that gut microbes promote synthesis of dopamine in guts. Moreover, in agreement with the role of dopamine in suppressing iNKT cell functions *in vivo* (Figure 1C), clearance of gut microbes significantly elevated CD69 expression and IFNγ production in hepatic iNKT cells after Con A injection (Figures 8A,B). Furthermore, increased ALT and AST (Figure 8C), and exacerbated hepatocyte necrosis (Figure 8D) were observed in these mice without gut microbiota. Consistent with the recovery of intestinal tyrosine hydroxylase after co-housing (Figures 7C,D), co-housed mice reduced IFNγ production in hepatic iNKT cells and showed ameliorated liver damage in response to Con A (Figures 8B–D). Overall, in agreement with their role in promoting synthesis of peripheral dopamine, gut microbes suppress iNKT cell-mediated liver damage. Notably, in antibiotics-treated mice, replenishment of dopamine D1-like receptor agonist A77636 significantly reduced Con A-induced liver injury, as indicated by decreased IFNγ production in hepatic iNKT cells, diminished ALT and AST level, and improved histological damage (Figures 7B–D). These results further confirm that reduced dopamine synthesis in microbiota-cleared mice contributed to the severer iNKT cell-mediated liver injury.

## Discussion

Dopamine has been previously shown as an immune modulator. Distinct effects of dopamine on T cells have been reported, and those are mediated through different subtypes of receptor (29, 30). It has been reported that activation of D1-like receptors inhibits suppressive functions of regulatory T cells, and promotes differentiation of T helper 2 (Th2) and especially T helper 17 (Th17) in naïve CD4^+^ T cells (31). Dopamine released by dendritic cells increases their IL12 and IL23 production through DRD5 in an autocrine manner, and consequently promotes Th17 differentiation (32), which contributes to inflammatory bowel diseases and multiple sclerosis. On the other hand, some studies report an inhibitory effect of dopamine on proliferation and cytotoxicity of CD4^+^ and CD8^+^ T cells through D1-like receptors (33). Interestingly, stimulation of DRD2 and DRD3, respectively elevates IL10 and TNFα production in resting human T cells (29), whereas activation of DRD3 reduces IL4 and IL10 mRNA but increases IFNγ mRNA in human activated CD4^+^ and CD8^+^ T cells (30). In addition to shaping T cell differentiation and function, dopamine could trigger T cell quiescence through DRD4, and that suggests a new way to treat lupus based on the higher expression of DRD4 in systemic lupus erythematosus patients (34, 35). Here, we show that dopamine represses IL4 and IFNγ production through D1-like receptors in iNKT cells (Figure 3B). D1-like receptors are coupled to G_s_, which increases cAMP concentration via activating adenylyl cyclase, whereas D2-like receptors are coupled to G_i_, which inhibits adenylyl cyclase and reduces intracellular cAMP (21). Although agonists of D1-like receptors could not distinguish DRD1 from DRD5, we detected expression of DRD5 protein but not DRD1 protein in iNKT cells (Figure 3E), indicating a predominant role of DRD5 in mediating suppressive effect of dopamine. Although mRNA of DRD3 and DRD4 were detected in iNKT cells, their receptor agonists showed no effect on cytokine production. It is possible that protein levels of these receptors are not correlated with mRNA levels. Moreover, we show that dopamine inhibits cytokine production in iNKT cells by activating cAMP-PKA pathway downstream DRD5. It has been previously shown that PKA activates CSK and therefor inhibits Lck activation (24, 25). We do not exclude this possibility. Additionally, our results indicate that activation of PKA by dopamine could inhibit cytokine responses in iNKT cells in a TCR proximal signaling independent manner. When iNKT cells were activated by PMA plus ionomycin bypassing TCR signaling, activation of PKA significantly inhibited NFκB activation (Figure 4F), which is a key transcriptional factor regulating iNKT cell functions (36, 37).

As an important neurotransmitter, dopamine has been critically linked to major depressive disorder. Lower amount of dopamine has been detected in patients suffering from depression (38). On the other side, the potential association between depression and chronic liver diseases, such as alcoholic liver disease and non-alcoholic fatty liver disease, has been suggested by several studies (39). Chronic liver disease patients show more severe depressive tendencies (40). Additionally, influences of depression on non-alcoholic fatty liver disease have also been reported, and non-alcoholic fatty liver disease patients with depression show worse therapeutic outcomes (41). It is possible that dopamine could be a mediator linking depressive disorder to liver diseases. As important liver resident cells, pathogenic roles of iNKT cells have been demonstrated during the development of non-alcoholic fatty liver disease, alcoholic liver disease, and autoimmune liver disease (8, 42, 43). Here, we show suppressive effects of dopamine on IL4 and IFNγ production from hepatic iNKT cells, and demonstrate a protective role of dopamine in inhibiting iNKT cell-mediated autoimmune hepatitis. Our results are consistent with previous findings that IFNγ plays critical roles in Con-A induced liver hepatitis (20), and IL4 promotes iNKT cell-mediated liver injury via an autocrine fashion (18). According to previous studies, dopamine regulates other immune cell functions as well (44). However, those effects would be minor in our models, as indicated by the results that A68930 failed to improve Con A-induced liver injury in *J*α*18*^−/−^ mice (Figure 5E). Overall, our studies suggest that suppressive effect of dopamine on cytokine production in iNKT cells would help to keep the immune tolerance in liver, and reduction of dopamine might contribute to the liver injury.

Although dopamine is a neurotransmitter, it could be synthesized peripherally. We detected tyrosine hydroxylase in guts, which is in agreement with previous findings that high amount of dopamine is detected in hepatic portal vein (6). According to previous studies, multiple cells in liver are able to produce dopamine, including dendritic cells, regulatory T cells, B cells, macrophages, and autonomic nervous system (5). However, small intestines expressed much higher amount of tyrosine hydroxylase than livers (Figure 7A). These results indicate that guts are main source of dopamine for regulating liver immunity, and explain the findings that although antibiotics reduced expression of tyrosine hydroxylase in small intestines but not in livers (Figure 7D), they significantly inhibited Con A-induced cytokine production in hepatic iNKT cells (Figure 8B). Gut microbiota has been previously shown to promote liver diseases via activating TLRs, modulating choline metabolism, and altering gut bile acids (45, 46). Conversely, our results (Figures 8C,D) and previous studies with bacteria free mice indicate a protective role of gut microbes in inhibiting liver injury (47). In addition to long chain fatty acids that have been shown to be synthesized by gut bacteria and reduce liver damage (48), we demonstrate a role of peripheral dopamine, synthesis of which is promoted by gut microbes, in suppressing liver injury. Due to the clearance of gut microbiota in our experiments, we could not exclude the possibility that some strains of bacteria might positively regulate the synthesis of dopamine, whereas other strains might regulate negatively. Moreover, it is still unclear whether the dopamine could be directly produced by gut microbes. It is rational to expect that dysbiosis of gut microbiota would influence biosynthesis of peripheral dopamine and consequently contributes to liver diseases.

In summary, we demonstrate that peripheral dopamine controlled by gut microbes inhibits IL4 and IFNγ production in iNKT cells and suppresses iNKT cell-mediated hepatitis. This microbes-dopamine-autoimmune hepatitis regulatory axis has to be further confirmed in human patients, and might provide new insight for clinical treatments.

## Author contributions

RX, HZ, JP, ZD, and WZ performed experiments. LB, RX, HZ, ZT, RZ, and ZZ designed the experiments. LB, RX, and HZ analyzed the data, prepared the figures, and wrote the manuscript. All authors read, commented, and approved final version of manuscript.

### Conflict of interest statement

The authors declare that the research was conducted in the absence of any commercial or financial relationships that could be construed as a potential conflict of interest.
