# Supplementary material for: Peripheral Dopamine Controlled by Gut Microbes Inhibits Invariant Natural Killer T Cell-Mediated Hepatitis
Source: Front Immunol. 2018 Oct 17;9:2398. doi: 10.3389/fimmu.2018.02398 (PMC6199378; doi:10.3389/fimmu.2018.02398)

**Figure S1. Gating strategy and purity of sorted iNKT cells**

iNKT cells were sorted from livers GFP^hi^ cells in *Vα14* *Tg.cxcr6^gfp/+^* mice. The purity of iNKT cells was about 90%. Data are representative of three independent experiments. Abbreviation: GFP, green fluorescent protein.

**
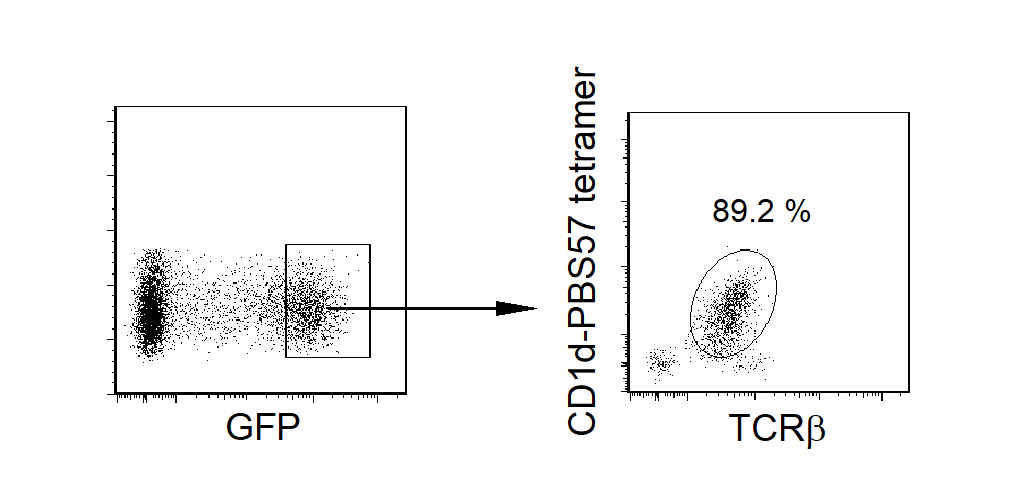
**

**Figure S2. MPTP reduces mRNA of *Th* in small intestines.**

mRNA level of *Th* in small intestines from control or MPTP treated mice (n = 4 mice per group). Error bars represent SEM. *P < 0.05. Abbreviation: MPTP, 1-methyl-4-phenyl-1,2,3,6-tetrahydropyridine; TH, tyrosine hydrogenase.


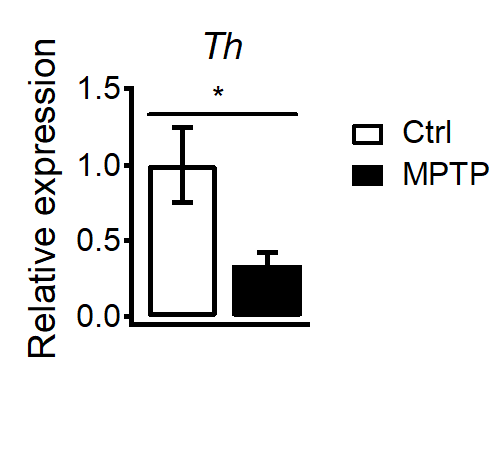


**Figure S3. Cell viability of iNKT cells.**

**(A-C)** Release of LDH in supernatants of iNKT cells in the presence of indicated reagents. Data are representative of three independent experiments. Abbreviation: DA, dopamine; LDH, lactate dehydrogenase.


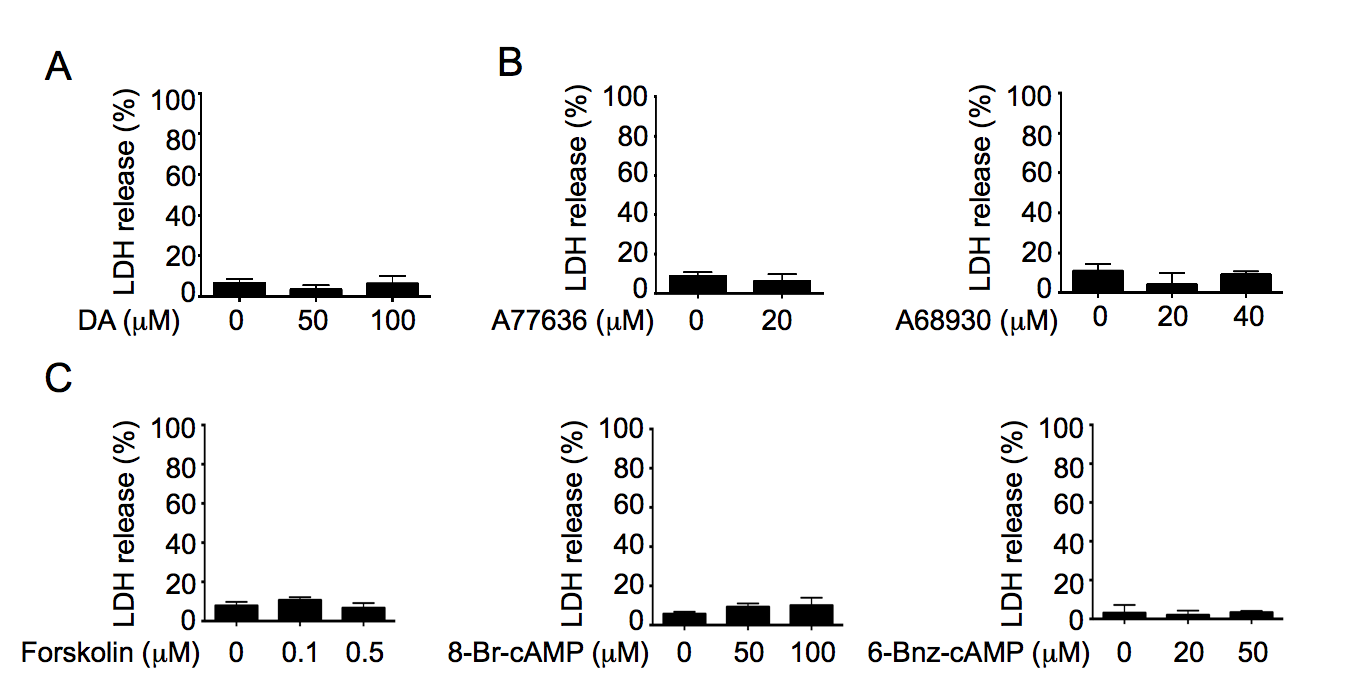

Supplement: Supplementary file 1 [file Data_Sheet_1.docx]
